# The Recent Progress of Tricyclic Aromadendrene-Type Sesquiterpenoids: Biological Activities and Biosynthesis

**DOI:** 10.3390/biom14091133

**Published:** 2024-09-07

**Authors:** Xiaoguang Yan, Jiaqi Lin, Ziming Liu, Sichone Daniel David, Dongmei Liang, Shengxin Nie, Mingyue Ge, Zhaohui Xue, Weiguo Li, Jianjun Qiao

**Affiliations:** 1 School of Chemical Engineering and Technology, Tianjin University, Tianjin 300072, China6222000029@tju.edu.cn (S.D.D.);; 2 Zhejiang Institute of Tianjin University, Shaoxing 312300, China; 3 Key Laboratory of Systems Bioengineering (Ministry of Education), Tianjin University, Tianjin 300072, China

**Keywords:** aromadendrene-type sesquiterpene, biological activity, biosynthesis

## Abstract

The tricyclic-aromadendrene-type sesquiterpenes are widely distributed and exhibit a range of biological activities, including anti-inflammatory, analgesic, antioxidant, antibacterial, insecticidal and cytotoxic properties. Several key sesquiterpene synthases (STSs) of this type have been identified, of which, viridiflorol synthase has been engineered for efficiently biosynthesizing viridiflorol in an *Escherichia coli* strain. This paper comprehensively summarizes the distribution and biological activity of aromadendrene-type sesquiterpenes in plant essential oils and microorganisms. The progress in aromadendrene-type sesquiterpene biosynthesis research, including the modifications of key STSs and the optimization of synthetic pathways, is reviewed. Finally, the prospects and associated challenges for the application and biosynthesis of these natural products are also discussed.

## 1. Introduction

Terpenoids are a large class of secondary metabolites mainly produced by plants and microorganisms in response to changes in the surrounding environment. They have complex chemical structures and conformations, and more than 80,000 terpenoids have been discovered [1]. They have been widely used in products such as biopharmaceuticals [2,3], natural pesticides and antibacterial agents [4], biofuels [5], perfumes and flavorings [6]. Sesquiterpenoids, constituting a significant group of terpenoids characterized by a C15 fundamental structure, are prominent among diverse compounds. The C15 skeleton compound is chemically modified by oxidation, acylation and glycosylation, forming various sesquiterpene alcohols, lactones and glycosides like artemisinin, celangulin V, nerolidol glucoside and bilobalide [3,4,7,8]. Investigating their presence in living organisms, their roles in biological processes and their efficient biosynthesis are currently key areas of scientific study.

**Figure 1:**
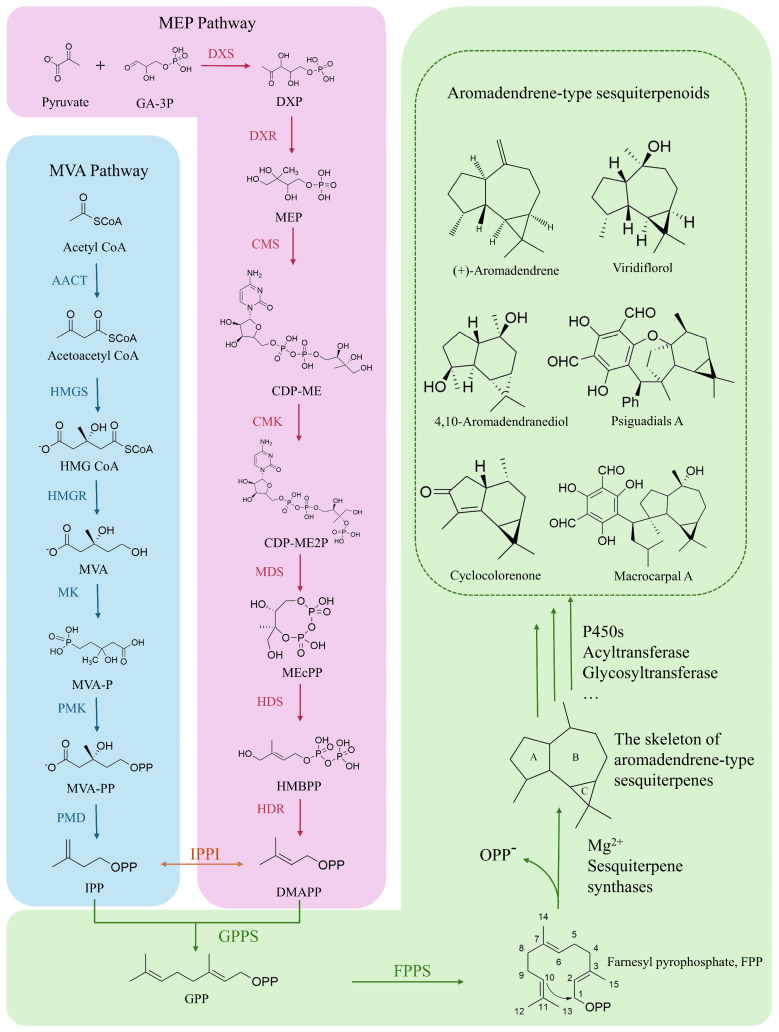
Biosynthesis pathways of aromadendrene-type sesquiterpenoids. Abbreviations: AACT, acetyl-CoA C-acetyltransferase; HMGS, hydroxymethylglutaryl-CoA synthase; HMGR, hydroxymethylglutaryl-CoA reductase; MK, mevalonate kinase; PMK, phosphomevalonate kinase; PMD, diphosphomevalonate decarboxylase; IPPI, isopentenyl diphosphate delta-isomerase; DXS, 1-deoxy-D-xylulose-5-phosphate synthase; DXR, 1-deoxy-D-xylulose-5-phosphate reductoisomerase; CMS, 4-diphosphatidyl-2-C-methyl-D-erythritol synthase; CMK, 4-diphosphatidyl-2-C-methyl-D-erythritol kinase; MDS, 2-C-methyl-D-erythritol 2,4-cyclodiphosphate synthase; HDS, 4-hydroxy-3-methylbut-2-enyl diphosphate synthase; HDR, 4-hydroxy-3-methylbut-2-en-1-yl diphosphate reductase; GPPS, geranyl diphosphate synthase; FPPS, farnesyl pyrophosphate synthase.

The aromadendrene-type sesquiterpenes were initially discovered and characterized in the essential oil of eucalyptus. These compounds have a core structure comprising a five-membered (ring A), a seven-membered (ring B) and a three-membered (ring C) carbon ring skeleton (Figure 1). They feature oxidation, acylation and glycosylation at different positions, leading to various biological effects, including anti-inflammatory, analgesic, antioxidant, antibacterial, insecticidal and cytotoxic properties. Consistent with the biosynthesis of other sesquiterpenes, the most basic structural units required for the synthesis of the skeleton of aromadendrene-type sesquiterpenes are isopentenyl diphosphate (IPP) and its isomer dimethylallyl diphosphate (DMAPP), which are mainly involved in the mevalonate (MVA) metabolic pathway in the cytoplasm and the methylerythritol phosphate (2-C-Methyl-D-erythritol-4-phosphate, MEP) metabolic pathway in the plastid. Acetyl-CoA is used to synthesize IPP via the MVA pathway. Meanwhile, pyruvate and 3-phosphoglyceraldehyde synthesize DMAPP via the MEP pathway. IPP and DMAPP are connected by farnesyl diphosphate synthase (FPPS) to form the final substrate required for sesquiterpene synthesis, farnesyl diphosphate (FPP). The aromadendrene-type sesquiterpenes are formed with FPP as the substrate under the catalysis of sesquiterpene synthases (STSs). After structural modification by cytochrome P450 oxidase and other tailoring enzymes, the basal skeleton compounds are modified to form sesquiterpenoid natural products with diverse activities. This study reviewed the relevant literature from the past 20 years and summarizes the distribution, bioactivities, metabolic pathways, key synthetic enzymes and the biosynthesis of aromadendrene-type sesquiterpenes. Furthermore, it attracts attention for further in-depth understanding and the use of aromadendrene-type sesquiterpenes in various biological contexts.

## 2. Distribution Characteristics of Aromadendrene-Type Sesquiterpenes

**Figure 2:**
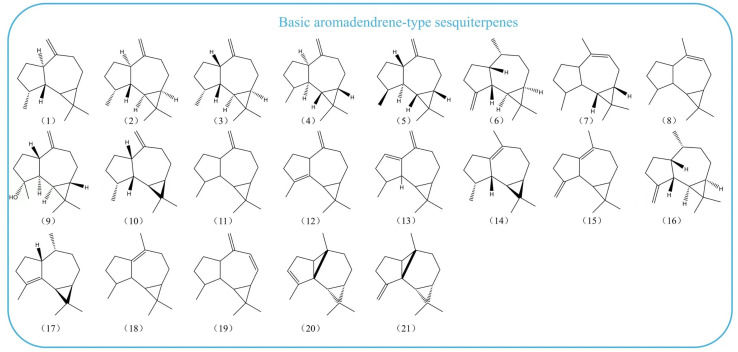

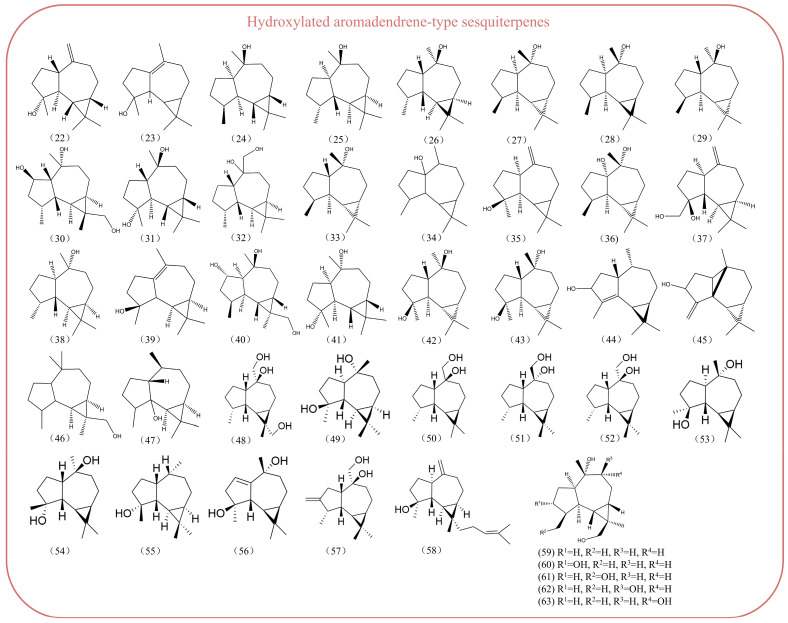

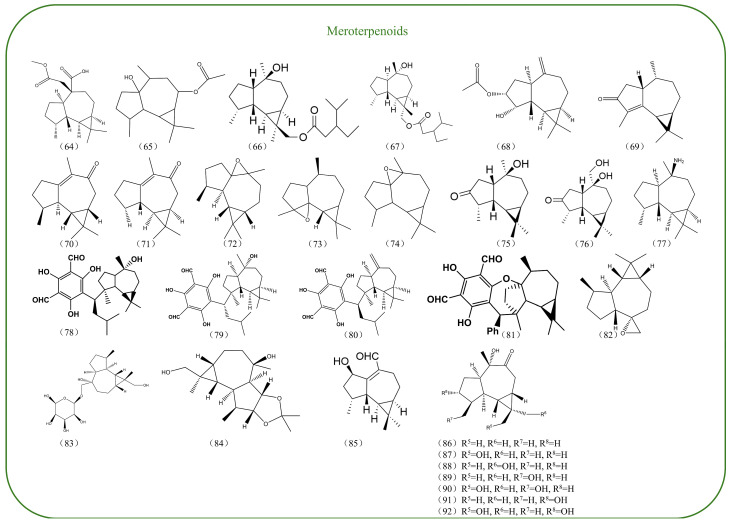
Structures of aromadendrene-type sesquiterpenoids. Compound name: α-aromadendrene (**1**), (+)-aromadendrene (**2**), (-)-alloaromadendrene (**3**), l-alloaromadendrene (**4**), β-diploalbicene (**5**), β-gurjunene (**6**), dehydroaromadendrene (**7**), alloaromadendr-9-ene (**8**), (1aS,4aS,7R,7aS,7bR)-1,1,7-trimethyl-4-methylene-1a,2,3,4a,5,6,7a,7b-octahydrocyclopropa[h]azulen-7-ol (**9**), alloaromadendrene (**10**), aromadendrene (**11**), aromadendra-4,10(14)-diene (**12**), (4α,5β,6α,7α,10α)-1-Aromadendrene (**13**), ledene (**14**), aromadra-1(10),4(15)-diene (**15**), β-seliene (**16**), α-gurjunene (**17**), viridiflorene (**18**), (-)-dehydroaromadendrene (**19**), anastreptene (**20**), myli-4(15)-ene (21), *ent*-spathulenol (**22**), (+)-isospathulenol (**23**), (+)-ledol (**24**), epi-globulol (**25**), viridiflorol (**26**), *ent*-viridiflorol (**27**), ledol (**28**), (-)-globulol (**29**), dichomitin B (**30**), *ent*-4β,10α-dihydroxyaromadendrane (**31**), (1aR,4aS,7R,7aS,7bS)-4-(hydroxymethyl)-1,1,7-trimethyl-2,3,4a,5,6,7,7a,7b-octahydro-1aH-cyclopropa[e]azulen-4-ol (**32**), (-)-epi-globulol (**33**), palustrol (**34**), spathulenol (**35**),cryophilain (**36**),(1aR,4aR,7S,7aR,7bR)-7-(hydroxymethyl)-1,1-dimethyl-4-methylidene-1a,2,3,4a,5,6,7a,7b-octahydrocyclopropa[h]azulen-7-ol (**37**), globulol (**38**), isospathulenol (**39**), 2β,13-dihydroxyledol (**40**), aromadendrane-4,10-diol (**41**), (-)-4a,7a-aromadendranediol (**42**), (-)-4b,7a-aromadendranediol (**43**), cyclocolorenol (**44**), myliol (**45**), flourensadiol (**46**), 5-hydroxy-α-gurjunene (**47**), allo-aromadendrane-10β,13,14-triol (**48**), 4β,10α-dihydroxyaromadendrane (**49**), (+)-10β,14-dihydroxy-allo-aromadendrane (**50**), allo-aromadendrane-10α,14-diol (**51**), allo-aromadendrane-10β,14-diol (**52**), aromadendrane-4β,10α-diol (**53**), alloaromadendrane-4α,10β-diol (**54**), 1,1,4,7-tetramethyldecahydro-1H-cyclopropa[e]azulen-7-ol (**55**), lochmolin F (**56**), allo-aromadendrene-10β,14α-diol (**57**), 4β-hydroxy-15-(3-methyl-2-butenyl)-aromadendra-10(12)-ene (**58**), inonotins H (**59**), inonotins I (**60**), inonotins J (**61**), inonotins K (**62**), inonotins L (**63**), (1aR,4S,4aR,7R,7aR,7bS)-4-(2-methoxy-2-oxoethyl)-1,1,7-trimethyl-2,3,4a,5,6,7,7a,7b-octahydro-1aH-cyclopropa[e]azulene-4-carboxylic acid (**64**), palustrol acetate (**65**), dysosesquiflorin A (**66**), dysosesquiflorin B (**67**), 3-acetoxyspathulenol (**68**), cyclocolorenone (**69**), millecrone B (**70**), squamulosone (**71**), ledene oxide (**72**), γ-gurjunenepoxide (**73**), ledene oxide-(II) (**74**), 3-oxo-10-alloaromadendranol (**75**), dysodensiol F (**76**), halichonadin F (**77**), macrocarpal A (**78**), macrocarpal B (**79**), macrocarpal C (**80**), psiguadials A (**81**), aromadendrene oxide 2 (**82**), dendroside A (**83**), sesquiterpenoid 2 (**84**), turranin F (**85**), inonotins A (**86**), inonotins B (**87**), inonotins C (**88**), inonotins D (**89**), inonotins E (**90**), inonotins F (**91**), inonotins G (**92**).

Most of the reported sesquiterpenes are derived from plant essential oils, with some derived from microbial fermentation products. A compound structure survey was carried out using the PubChem database. We searched chemicals by the basic aromadendrene-type structure and filtered the candidate compounds without literature information. Based on the varying degrees of modification in the skeletal structure, we classified and exhibited the aromadendrene-type sesquiterpenes into three categories: the basic and the hydroxylated aromadendrene-type sesquiterpenes and the meroterpenoids (Figure 2).

### 2.1. Aromadendrene-Type Sesquiterpenoids in Plant Essential Oils

Plant essential oils are predominantly mixtures of various terpenoids in different proportions and exhibit a wide range of bioactivities, especially antimicrobial, insecticidal and cytotoxic properties. Therefore, plant essential oils are widely used in the flavor, fragrance, pharmaceutical and cosmetic fields. Aromadendrene-type sesquiterpenes are commonly found in the essential oils of many plants and have various biological activities. The quality and chemical composition of plant essential oils vary, as reported in the existing research literature, due to differences in living environments, harvesting parts and plant preparation processes [9]. This part overviews the plant essential oils containing a relatively high concentration of aromadendrene-type sesquiterpenes in their components. As indicated in Table 1, plants from various genera and families have been found to contain the aromadendrene-type sesquiterpenes with diverse structures. The high levels of sesquiterpenes such as compound (**26**), ledol (**28**) and compound (**17**), were notably identified in the plant essential oils. A certain amount of compound (**26**) was found in the essential oils of the Lamiaceae family plants. Especially in *Salvia algeriensis*, the compound (**26**) content was 71.1%, which is relatively higher than the other components. Compound (**26**) is known for its evident anti-inflammatory, antioxidant and antimicrobial (especially anti-*Mycobacterium tuberculosis*) activities [10]. Additionally, compound (**26**) is a fragrance molecule used to develop personal-care products [11]. Thus, compound (**26**) has the potential for application in the agriculture and food industry. In-depth research on the distribution, synthesis regulation mechanism and activity analysis of aromadendrene-type sesquiterpenes in specific species of plants will improve the application scenarios of such compounds.

### 2.2. Aromadendrene-Type Sesquiterpenoid in Microorganisms

Most terpenoids used in industrial applications are derived from plants, although such compounds are also widely found in bacteria and fungi. Recently, fungal terpenoids, especially the sesquiterpenoids from Basidiomycetes, have received special attention. The basidiomycete *Agrocybe aegerita* is rich in sesquiterpenoids. A total of 31 sesquiterpenoids were detected when *A. aegerita* was cultured to the sporulation stage, including three aromadendrene-type sesquiterpenes, viridiflorene (**18**), compound (**26**) and *β*-gurjunene (**6**) [38]. Furthermore, aromadendrene-type sesquiterpenoids have also been identified in fungi such as *Muscodor yucatanensis*, *Aspergillus aculeatus* lzuka, *Penicillium melinii* Thom and *Monascus purpureus* [39,40,41]. The discovery of these compounds has laid a foundation for further research on the distribution and synthesis mechanism of aromadendrene-type sesquiterpenoids in microorganisms.

## 3. Biological Activities of Aromadendrene-Type Sesquiterpenes and Their Related Plant Essential Oils

This kind of sesquiterpene has been shown to have biological effects such as anti-inflammatory, analgesic, antioxidant, antibacterial, insecticidal and cytotoxic properties. Research on these sesquiterpenes, found mainly in plant essential oils, has extended their potential applications.

### 3.1. Anti-Inflammatory and Analgesic Activities

Aromadendrene-type sesquiterpenes, such as compound (**26**) and spathulenol (**35**), have broad anti-inflammatory activities. *Allophylus edulis* is an edible and medicinal plant from South American and is widely found in Brazil. Its leaf extract is commonly used in folk treatments for its anti-inflammatory and antioxidant potential. In particular, the extract is associated with a popular treatment for diabetes. The essential oil obtained from the leaves of *A. edulis* contained 30.88% of compound (**26**). The essential oil, as well as compound (**26**), exhibited promising anti-arthritic effects by alleviating mechanical hyperalgesia, reducing swelling (edema), lowering the total leukocyte count, decreasing polymorphonuclear cell numbers, inhibiting nitric oxide production and minimizing protein leakage in models of zymosan-induced joint inflammation [34]. Compound (**26**) also showed analgesic properties in formalin-induced pain sensitivity tests and in experiments that induced hyperalgesia. It may significantly inhibit the metabolism of tumor necrosis factor-*α* (TNF-*α*) and dopamine (DOPA) and underlie the anti-hyperalgesic attributes of *A. edulis* essential oil. Furthermore, this anti-inflammatory and pain-relieving potential might partially justify the traditional use of *A. edulis* as a treatment for pain. The essential oil extracted from the leaves of *Psidium guineense* contained 38 components, of which compound (**35**) occupied the dominant proportion (80.7%) [20]. Oral administration of the essential oil and compound (**35**) showed significant inhibition in the Cg-induced mice paw oedema and pleurisy model, with GI (50) values ranging from 0.89 to 49.30 μg/mL, indicating that they may be particularly effective against the ovarian cancer cell line. Furthermore, both showed antioxidant activities in the DPPH and MDA system, with IC _(50)_ values ranging from 26.13 to 85.60 μg/mL compared with the reference standard. In addition, they also exhibited antimycobacterial activity. The essential oil components of three species of Lamiaceae plants in Ecuador, *Lepechinia heteromorpha* (Briq.) Epling, *Lepechinia radula* (Benth.) Epling and *Lepechinia paniculata* (Kunth) Epling, mainly contained sesquiterpenoids, which made up about 62.8% of the total components. The primary constituents were found to be compound (**18**) (27.3%) and compound (**28**) (21.2%) [12]. These essential oils are commonly used locally to alleviate headaches and other neurological conditions. *Melaleuca alternifolia* (also known as the Australian tea tree) is a plant in the Myrtaceae family. Its essential oil has been proven to be very effective in controlling various parasitic infections. It primarily consists of monoterpenes like terpinen-4-ol, as well as a certain amount of aromadendrene-type sesquiterpenes, including aromadendrene (**11**), compounds **18** and **26** and globulol (**38**). Their effectiveness in combating parasitic infections is largely due to their antihistamine and anti-acetylcholinesterase properties and their ability to regulate the host’s inflammatory response [19].

### 3.2. Antibacterial and Insecticidal Activity

Volatile terpenoids function predominantly as inherent protective mechanisms that are released in substantial quantities from plants in response to attacks by pathogenic microorganisms or pests [42]. These compounds are mainly toxic to fungi, bacteria and viruses [43]. Further comprehensive research into volatile terpenoids may lead to the development of compounds that can protect crops against agricultural fungal infections or pests in the future. Two Eucalyptus plants (*E. microtheca* and *E. viminalis*) that are pharmacologically potent against diabetes, hepatotoxicity and inflammation contain various sesquiterpenoids, including compound (**11**) (12.773%), compound (**38**) (3.054 to 5.997%) and ledene (**14**) (5.665%) [18]. In addition, the petroleum ether fraction of its crude ethanol extract, mainly containing compound (**38**), exhibited effective inhibitory actions on *Fusarium graminearum*, *Rhizobium graminearum* and *Botrytis cinerea*. Furthermore, these plant essential oils could be an alternative source of natural insecticide agents to be used in medicinal and food products. *Syzygiella rubricaulis* is a kind of bryophyte that contains 50 compounds in its essential oil, with hydrocarbon sesquiterpenes (48.35%) and oxygenated sesquiterpenes (46.89%) being the predominant constituents [28]. The essential oil of this plant exhibited antibacterial, antioxidant and anticholinesterase activities Among these active effects, it showed a strong inhibitory effect on acetylcholinesterase (AChE), with an IC_50_ value of 26.75 ± 1.03 μg/mL, a moderate antibacterial effect (MIC 500 μg/mL) and antioxidant effects (ABTS: SC_50_ is 343.38, DPPH is 2650.23 μg/mL). These results suggested the potential therapeutic application of the bryophyte essential oil in treating Alzheimer’s disease due to its potent anticholinesterase properties. The chemical compositions of essential oils of *Rhododendron tomentosum* from eastern Lithuania at different stages were analyzed by GC-MS [44]. In total, up to 70 compounds were identified, among which palustrol (**34**) (24.6 to 33.5%) and compound (**28**) (18.0 to 29.0%) had the highest proportions. Moreover, the results of the antibacterial experiment on pathogenic yeast and the free radical scavenging activity experiment showed that the essential oil of this plant showed potential antibacterial and free radical scavenging abilities. *Xanthomonas citri* is a serious disease that affects the healthy growth of citrus plants worldwide, causing a disease referred to as citrus canker. The chemical composition and antibacterial activity of essential oils from the fresh and dried leaves of *Schinus molle L* were compared [45]. The subsequent analysis found that *S. molle* essential oil was rich in compound (**35**), which showed inhibition against *X. citri*. In another analysis the of the aromadendrene-type sesquiterpenoid aromadendrane-4,10-diol (**41**) isolated from the leaves of *Xylopia brasiliensis*, the compound exhibited inhibitory activity against the fungus *Cladosporium cladosporioides* [46]. These compounds can potentially inhibit this particular fungus’s growth, suggesting possible applications in drug development or agriculture. Compound (**26**), as the dominant compound in the essential oil of *Lepidozia chordulifer*, could inhibit bacterial growth and biofilm formation. Specifically, a 50 μg/mL concentration of compound (**26**) could inhibit 60% of *Pseudomonas aeruginosa* and 40% of *Staphylococcus aureus* [47]. The essential oil of *Eriocephalus africanus L.* in the Asteraceae family has compound (**28**) (19.92%) as one of the major components. This essential oil exhibited a certain inhibitory effect on tested pathogens, including *Agrobacterium tumefaciens*, *Dickeya solani*, *Erwinia amylovora*, *Pseudomonas cichorii* and *Serratia plymuthica* [48]. Among these, the antibacterial effect on *D. solani* was the most prominent. In the essential oils from three Ethiopian medicinal plants, *Hagenia abyssinica* (Rosaceae), *Leonotis ocymifolia* (Lamiaceae) and *Moringa stenopetala* (Moringaceae), compound (**28**) (58.57%) was the principal volatile oil component and showed trypanocidal activity [48].

### 3.3. Antioxidant Activity and Cytotoxicity

Antioxidant activity and cytotoxicity are usually correlated [49,50], and some aromadendrene-type sesquiterpenes have shown potential antioxidant and cytotoxic properties. The essential oils from the leaves of *Cinnamomum osmophloeum*, a native tree species in Taiwan, exerted in vivo antioxidant activities on *Caenorhabditis elegans* [25]. Subsequent research revealed that a key component of the essential oil, alloaromadendrene (**10**), played a significant role in juglone-induced oxidative stress in *C. elegans.* Furthermore, compound (**10**) not only protects against oxidative stress but also extends the lifespan of *C. elegans* by affecting the FOXO transcription factor DAF-16. These results suggested that compound (**10**) and the leaf essential oil of *C. elegans* hold promise as sources for antioxidants or therapies to delay aging. The components and cytotoxicity of the essential oils of *Eryngium campestre* and *Eryngium amethystinum* were compared [33]. The study found that both essential oils were rich in sesquiterpenoids, including compounds **28** and **35**. Both plant essential oils showed strong cytotoxicity to tumor cells, with IC_50_ values of 1.65–5.32 and 1.57–2.99 μg/mL for *E. amethystinum* and *E. campestre*, respectively, which are comparable to the anticancer drug cisplatin. Additionally, *E. amethystinum* essential oil also exhibited antioxidant activity. Compound (**26**) may have anticancer properties at different concentrations (ranging from 0.03 to 300 μM) on three types of cancer cells in vitro: breast cancer (MCF-7), lung cancer (A549) and brain cancer (Daoy) [51]. This would provide a potential therapeutic option for patients. *Garcinia quaesita* and *Garcinia zeylanica* are plants endemic to Sri Lanka and have certain medical and health care effects. These two plant essential oils contained 12.12% of compound (**11**) and demonstrated significant antioxidant activities [29].

### 3.4. Other Activities

Aromadendrene-type sesquiterpenes have also shown other biological activities. Some novel compounds, particularly aromadendrane-4β,10α-diol (**53**), were screened using an NGF-induced PC12 differentiation model for the potential to induce neuronal differentiation [52]. The results showed that compound (**53**), identified from the dried twigs of the Baccharis gaudichaudiana, exhibited the potential to promote neurite outgrowth in neuronal cells in vitro. Compound (**53**) effectively enhanced neurite outgrowth in NGF-treated PC12 cells and the N1E115 cells in a time-dependent manner. In cultured primary cortical neurons, compound (**53**) significantly increased neurite outgrowth and magnificently increased the number of neurites on the soma and bifurcations. Further studies demonstrated that compound (**53**) was active in inducing neurite growth by activating the ERK signaling pathway, which may be beneficial in treating brain diseases. A study of ethyl acetate extract from Aristolochia yunnanensis, a traditional Chinese medicine, found that the active extract has therapeutic effects in vitro and in vivo on myocardial fibrosis, which causes systolic and diastolic dysfunction in many cardiac physiologies. Antifibrotic therapy may be a key strategy to curb many fibrosis-related heart diseases. Ten sesquiterpenoids, including compound (**35**) and (+)-isobicyclogermacrenal, were isolated from active extracts of Aristolochia and their anti-fibrotic effects were evaluated using the transforming growth factor β 1 (TGFβ1)-stimulated cardiac fibroblasts and NIH3T3 fibrosis model [53]. The results showed that the main active ingredients, compound (**35**) and (+)-isobicyclogermacrenal, were more effective than the well-known natural anti-fibrotic agent oxymatrine. Further study proved that the aromadendrene-type sesquiterpene, compound (**35**), could inhibit TGFβ1-induced cardiac fibroblast proliferation by downregulating mRNA levels and inhibiting the expression of fibrosis biomarkers fibronectin and α-smooth muscle actin. It also revealed that this compound could stop the activation of the TGFβ type I receptor, leading to reduced activation of Smad2/3, which, in turn, prevents the movement of Smad2/3 into the cell nucleus in the TGFβ/Smad signaling pathway. These findings suggested that compound (**35**) could serve as a lead compound for the development of drugs to combat myocardial fibrosis. Cyclocolorenone (**69**) and its supposed precursor compound (**17**), which have phytotoxic and antimicrobial properties, were identified from *Solidago canadensis*. This type of sesquiterpenoid is a fixative in the perfume industry [54].

## 4. Synthetic Biology Research on Aromadendrene-Type Sesquiterpenes

The terpenoids produced in natural plants or microbial hosts are low in content and complex in structure, making them challenging to synthesize efficiently. At present, using metabolic engineering and synthetic biology methods can precisely and efficiently synthesize specific terpenoids in engineered microbial hosts, and this has become a hot topic in the research of natural product biosynthesis. Generally, strategies like the efficient screening of key synthetic enzymes and protein engineering, heterologous remodeling of metabolic pathways, regulation of host metabolism and subcellular localization of specific metabolism can be combined to achieve the efficient biosynthesis of target terpenoid products. In the past decade, many natural sesquiterpenoids, such as compound (**26**), santalol, patchoulol and nerolidol, have been efficiently synthesized in heterologous hosts [11,55,56,57]. In addition, the microbial synthesis of some terpenoids, such as farnesene and artemisinin acid [58,59], has made breakthroughs in industrial-scale production.

### 4.1. Discovery and Modification of Key Enzymes for the Synthesis of Aromadendrene-Type Sesquiterpenes

Key synthetic elements such as STSs are a bottleneck for the efficient biosynthesis of sesquiterpenes. Although metabolic engineering can be used to rationally strengthen the upstream pathway for producing sesquiterpenes, namely, reinforcing the acetyl-CoA synthetic pathway, by enhancing the metabolic flow from acetyl-CoA to FPP and slightly inhibiting the downstream substrate competition pathway can the heterologous biosynthesis of target sesquiterpenes be initially achieved. However, in the later stage of transformation, the excessively enhanced secondary metabolic flow increases the growth burden of the engineered strain and the yield of the target compound reaches a bottleneck period. At this time, it is important to achieve efficient production of sesquiterpenes by exploring synthetic elements such as STSs with superior performance and improving their catalytic characteristics through QM/MM, MD techniques, machine learning and high-throughput screening of mutants to break through the yield bottleneck. The currently reported aromadendrene-type STSs include viridiflorol synthase, viridiflorene synthase and α-gurjunene synthase (Table 2) [38,60]. Some other STSs can also catalyze the formation of byproducts that belong to aromadendrene-type sesquiterpenes, like compounds **11**, **28**, **35** and **38**. The majority of synthases engaged in the specific synthesis of aromadendrene-type sesquiterpene remain undocumented to date.

At present, many successful cases of improving the performance of STSs have been obtained by changing the catalytic activity and specificity of enzymes through irrational and rational modified methods [70,71,72,73,74]. Based on homologous sequence alignment, the viridiflorol synthase (VS) from *Agrocybe aegerita* showed an additional 80 amino acids in the N-terminus compared with other closely related fungal terpene synthases. These extra amino acids could be a signal peptide, which may be related to plasma membrane localization and expression of terpene synthases in organisms, thereby reducing the contact with the enzyme substrate in the cytosol [11]. Combined with the error-prone PCR random mutations, the VS mutant was truncated by 80 amino acids at the N-terminus and the compound (**26**) yield increased by three times. According to the literature, bicyclogermacrene may be an important intermediate in the synthesis of sesquiterpenes with antibacterial activity, such as compounds **11**, **26**, **28**, **34** and **38**. Moreover, it may also be an important synthetic skeleton for some meroterpenoids with antibacterial activity, such as psiguadials A (**81**) and macrocarpal A (**78**) [75]. When investigating if bicyclogermacrene synthase is beneficial for the biosynthesis of aromadendrene-type sesquiterpenes, our research group discovered a novo STS (JeSTS4) from *Jungermannia exsertifolia* through transcriptome analysis. The main product generated by this enzyme was bicyclogermacrene, as well as compound (**26**) [76]. In addition, two hotspot regions of JeSTS4 were identified by the coevolution study and computational simulations. In particular, the G91S and R242K mutations improved the conversion rate of JeSTS4, which could improve its application potential. Recently, a new *ent*-viridiflorol synthase CryA was identified through genome mining of the Actinomycetes *Crossiella cryophila* [67]. Moreover, a new compound, cryophilain (**36**), was produced by CryA and the related P450 CryB. CryB could serve as a tailoring enzyme responsible for installing a distinctive bridgehead hydroxy group at a 5/7/3-fused tricyclic skeleton. These results enriched the terpene natural product library, expanded the tool enzyme library of P450s and promoted the research progress of aromadendrene-type sesquiterpene biosynthesis. Based on crystal structure, combined with computational methods such as QM/MM and machine learning, the catalytic mechanisms of STSs are gradually being unveiled. The generation and quenching of carbocation [77,78], the induced-fit mechanism of the enzyme cavity [70,71] and the plasticity residues affecting catalytic efficiency were well demonstrated. In particular, Allemann’s group redesigned the active pocket of some STSs to produce complex cyclic hydrocarbons like germacradien-11-ol [79,80,81,82], patchoulol [74] and selin-7(11)-en-4-ol [83] by simulation-guided engineering. However, the current analysis of the mechanism of STSs is not systematic enough, especially for aromadendrene-type STSs. The absence of the crystal structure of aromadendrene-type STSs greatly restrains the study of their catalytic mechanisms. A comprehensive analysis of the crystal structure and mechanism of STSs could significantly improve their redesign process, contributing to the efficient biosynthesis of aromadendrene-type sesquiterpenes.

### 4.2. Selection of Different Hosts for the Synthesis of Aromadendrene-Type Sesquiterpenes

Currently, model microorganisms such as *Saccharomyces cerevisiae* and *Escherichia coli*, as well as non-conventional microorganisms such as *Pichia pastoris*, *Yarrowia lipolytica* and *Hansenula polymorpha* and plant hosts such as tobacco, have been used to synthesize sesquiterpenoids, each having its unique advantages. The model strains *E. coli* and *S. cerevisiae* have a clear genetic background and are easy to culture. The genetic tools for their metabolic engineering are relatively mature, and they also have precursor metabolic pathways for sesquiterpenoid synthesis, making them ideal hosts for the heterologous synthesis of sesquiterpenoids. Currently, these two model microorganisms have been transformed into cell factories to efficiently synthesize terpenoid compounds such as compound (**26**), sclareol and limonene [11,84,85]. An engineered *E. coli* strain for the efficient synthesis of compound (**26**) was optimized through a comprehensive strategy including transcriptional regulation, metabolic engineering and enzyme engineering [11]. The optimized strain produced 25.7 g/L and a yield of 0.22 g of compound (**26**)/g of glucose in 2.5 days, which is the highest yield of this product reported so far. However, it is difficult for *E. coli* to express eukaryotic enzymes such as P450s, which makes it difficult to produce various sesquiterpenoids that require post-oxidation. In comparison, *S. cerevisiae* has a eukaryotic expression modification system and is generally recognized as a safe (GRAS) strain suitable for the biosynthesis of various sesquiterpenoids.

The non-conventional strain *P. pastoris* has a rich metabolic pathway for cofactors. It is suitable for high-density culture and can naturally utilize cheaper fatty acids and methanol as carbon sources to produce natural products. With the development and optimization of gene editing tools, various sesquiterpenes, such as farnesene and santalene, have been efficiently synthesized in *P. pastoris* [86,87]. *Y. lipolytica* is a typical oleaginous yeast with a good supply of acetyl-CoA, a wide range of substrate adaptability and unique physiological characteristics, showing its great potential for synthesizing natural products. *Y. lipolytica* demonstrates broad pH adaptability and can thrive in diverse environments, including in environemts with pH values ranging from 3.5 to 8.0. This characteristic enhances its suitability for various fermentation conditions. Additionally, this yeast exhibits a wide range of substrate (glycerol, molasses, xylose, raw starch, cellobiose, cellulose or inulin) utilization capabilities and shows a strong capacity for lipid metabolism. Because of this, *Y. lipolytica* has been recently proposed as an excellent candidate for sesquiterpenoid biosynthesis, such as *α*-farnesene [88], *α*-humulene [89] and *α*-santalene [90]. However, its gene editing tools, such as genetic components and CRISPR/Cas9 systems, are not yet perfect and require further development.

In comparison to heterologous synthesis, hosts capable of synthesizing aromadendrene-type sesquiterpenes naturally are potentially more suitable for the high expression of inherent STSs with magnificent properties [91]. Furthermore, natural hosts have evolved dynamic regulation, special cell structures, like glandular trichomes [92], and efficient secretory system for sesquiterpene products, making them a more perfect choice for synthesizing related products. The essential oil of *Mentha piperita* is rich in a certain amount of compound (**26**) and has a wide range of applications in the cosmetic and pharmaceutical fields. The endogenous viridiflorol synthase gene *MpTPS4* was overexpressed in *M. piperita* under a tissue-specific promoter and a maximum of about 25% of compound (**26**) could be obtained in a transgenic plant. In addition, this transgenic plant possessed better growth. It produced essential oil of good quality, containing a high content of compound (**26**) and a proportionate decrease in the amounts of menthone, menthofuran and menthol compared to the other control genotype [69]. That would make it better for use in perfumes and cosmetic and pharmaceutical products.

### 4.3. Metabolic Engineering to Improve Sesquiterpenoid Production

**Figure 3:**
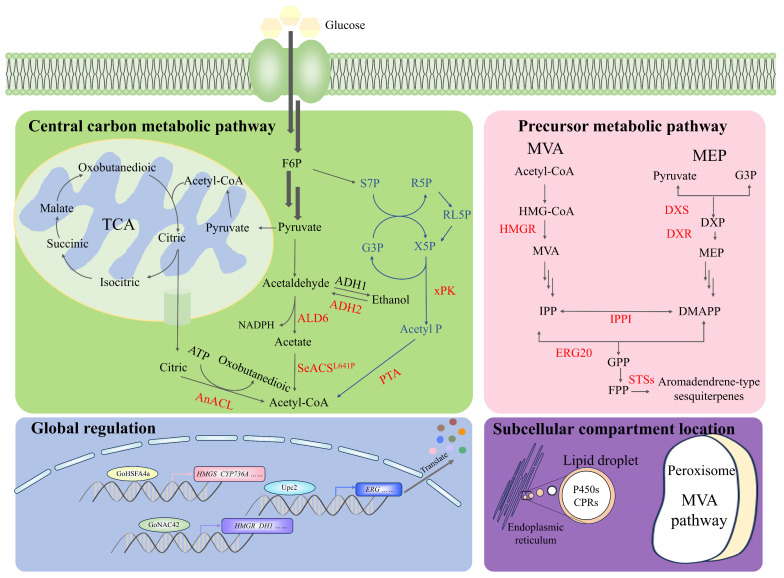
Metabolic engineering strategies to improve sesquiterpenoid production.

In order to increase the capability of producing sesquiterpenoids for hosts, a comprehensive strategy can be initially used to efficiently enhance metabolic flow (Figure 3). Firstly, rate-limiting enzymes in the precursor metabolic pathway of sesquiterpenoids need to be overexpressed or engineered (fusion, truncation and mutation) in the host cell. HMGR, IPPI and FPPS for the MVA metabolic pathway and DXS and IPPI for the MEP metabolic pathway should be preferentially considered. Moreover, the optimization of other steps in the MVA or MEP pathways might also be necessary to obtain higher sesquiterpenoid titers. In addition, screening key enzymes in the pathway from other organisms has been proven to be an advisable strategy to increase sesquiterpenoid titers [59,93]. Secondly, integrating alternative pathways to the precursor, such as the phosphoketolase pathway, or even reconstructing central metabolism, could potentially increase the supply of acetyl-CoA and the utilization rate of carbon sources, resulting in a significant efficiency for the synthetic production of sesquiterpenoids [93,94]. Thirdly, a global transcriptional influence on the metabolic pathways for sesquiterpenoid synthesis is crucial for optimizing gene expression, balancing metabolic flux, increasing terpenoid tolerance and inducing efflux, which can improve the production of target terpenoids [95,96,97,98]. In addition, subcellular compartments, such as the mitochondria, peroxisome and endoplasmic reticulum, have also been chosen as the location of sesquiterpenoid production pathways to facilitate magnificent yield improvements [99]. Cellular environments with unique properties should be considered for targeting a specific biosynthetic pathway. The compartmentalization strategy also enhances substrate concentration, contributing to the conversion by heterologous enzymes and reducing the toxicity effects of hydrophobic intermediates accumulating intracellularly.

At present, metabolic engineering modification of hosts for the synthesis of aromadendrene-type sesquiterpenes has only been reported in *E. coli*. Namely, the MVA metabolic pathway of *S. cerevisiae* was engineered into *E. coli*, leading to a comprehensive enhancement of compound (**26**) production. Based on the systematic coordination of the transcription of the sesquiterpenoid metabolic pathway and the improvement of the translation of the viridiflorol synthase with optimized properties, the highest yield of compound (**26**) was obtained [11]. Combining a variety of strategies to engineer and screen hosts suitable for synthesizing target products will pave the way for the commercialization of aromadendrene-type sesquiterpene production.

## 5. Conclusions

Aromadendrene-type sesquiterpenoids are widely distributed in plants and microorganisms and their tricyclic structures with oxidation and other modifications generated a lot of novel compounds. Some reported aromadendrene-type sesquiterpenoids, such as compounds **26**, **28** and **35**, possessed anti-inflammatory, antioxidant, antibacterial, insecticidal and cytotoxic properties. At present, the distribution, synthetic regulation mechanisms and specific activities of such compounds need to be further studied to improve their application scenarios.

With the development of synthetic biology and the improvement of sequencing technology, more and more sesquiterpenoids have achieved efficient heterologous synthesis. This new production pattern will become a strong competitor and substitute for the traditional production model. Although there are numerous aromadendrene-type sesquiterpenes, most of them have not been efficiently biosynthesized due to the lack of key STSs and the unknown catalytic mechanisms.

More and more aromadendrene-type STSs will be discovered by using multi-omics analysis, including high-resolution genome sequencing and spatiotemporal transcriptome and metabolome analysis. Integrating computational chemistry approaches and machine-learning-derived renovation methods, specific and efficient enzymes will be redesigned for the biosynthesis and extensive application of aromadendrene-type sesquiterpenes.

## Figures and Tables

**Table 1:** Aromadendrene-type sesquiterpenoids in plant essential oils or microorganisms ^a^.

Compound Name	Species	Family and Genus		Content (%)	Ref.
**Plants**					
Viridiflorene (**18**)	*Lepechinia heteromorpha*	Lamiaceae	Lepechinia	27.30%	[12]
Viridiflorol (**26**)	*Salvia algeriensis*		Salvia	71.10%	[13]
Viridiflorol (**26**)	*Satureja visianii*		Satureja	17.90%	[14]
Viridiflorol (**26**)	*Mentha aquatica*		Mentha	11.30%	[15]
Ledol (**28**)	*Lepechinia heteromorpha*		Lepechinia	21.20%	[12]
*epi*-Globulol (**25**)	*Teucrium montanum*		Teucrium		[16]
*α*-Gurjunene (**17**)	*Melaleuca alternifolia*	Myrtaceae	Melaleuca	Stem (1.1%)	[17]
Aromadendrene (**11**)	*Eucalyptus microtheca*		Eucalyptus	Leaves (12.773%), flowers (7.444%)	[18]
Aromadendrene (**11**)	*Melaleuca alternifolia*		Melaleuca	Stem (1.6%)	[19]
Viridiflorene (**18**)				Stem (1.6%)	[18]
Viridiflorol (**26**)				Stem (1.3%)	
Globulol (**38**)				Stem (1.4%)	
Globulol (**38**)	*Eucalyptus microtheca*		Eucalyptus	Leaves (5.997%), flowers (5.419%)	[18]
Globulol (**38**)	*Eucalyptus viminalis*			Leaves (3.054%)	[18]
Spathulenol (**35**)	*Psidium guineense*		Psidium	Leaves (80.7%)	[20]
*α*-Gurjunene (**17**)	*Mikania micrantha*	Asteraceae	Mikania	9%	[21]
Viridiflorol (**26**)	*Senecio rowleyanus*		Senecio	11.00%	[22]
Ledol (**28**)	*Eriocephalus africanus*		Eriocephalus	Leaves (19.92%)	[23]
Isospathulenol (**39**)	*Anthemis pignattiorum*		Anthemis	10.60%	[23]
Viridiflorene (**18**)	*Cryptocarya bellendenkerana*	Lauraceae	Cryptocarya		[24]
Alloaromadendrene (**10**)	*Cryptocarya osmophloeum*			Leaves (5.0%)	[25]
Viridiflorol (**26**)	*Cryptocarya bellendenkerana*				[24]
*α*-Gurjunene (**17**)	*Dimocarpus longan*	Sapindaceae	Dimocarpus	Peel (11–24%)	
Viridiflorol (**26**)	*Allophylus edulis*		Allophylus	Leaves (30.88%)	[26]
*α*-Gurjunene (**17**)	*Panax ginsengr*	Araliaceae	Panax	Root (8%)	[27]
Viridiflorene (**18**)	*Syzygiella rubricaulis*	Cryptocapsaceae	Syzygiella		[28]
Alloaromadendrene (**10**)	*Garcinia quaesita*	Lutaceae	Garcinia	Leaves (11.12%)	[29]
Ledol (**28**)	*Rhododendron tomentosum*	Ericaceae	Rhododendron		[30]
Palustrol (**34**)					
*β*-Gurjunene (**6**)	*Murraya koenigii*	Rutaceae	Murraya	25%	[31]
Isospathulenol (**39**)	*Murraya paniculata*				
Ledol (**28**)	*Hagenia abyssinica*	Rosaceae	Hagenia	Flowers (58.57%)	[32]
Ledol (**28**)	*Eryngium campestre*, *Eryngium amethystinum*	Apiaceae	Eryngium		[33]
Spathulenol (**35**)	*Eryngium campestre*, *Eryngium amethystinum*				
Spathulenol (**35**)	*Schinus molle*	Burseraceae	Schinus		[34]
Myli-4(15)-ene (**21**)	*Mylia taylorii*, *Mylia nuda*	Jungermanniaceae	Mylia		[35,36]
Aromadendrene (**11**)					
Aromadendra-4,10(14)-diene (**12**)					
*α*-Gurjunene (**17**)					
Anastreptene (**20**)					
Viridiflorene (**18**)					
Viridiflorol (**26**)					
Globulol (**38**)					
Myliol (**45**)					
Spathulenol (**35**)					
Aromadendrene (**11**)	*Jungermannia exsertifolia*	Jungermanniaceae	Jungermannia		[37]
Viridiflorol (**26**)					
**Microorganisms**					
Viridiflorene (**18**)	*Agrocybe aegerita*	Strophariaceae	Agrocybe		[38]
*β*-Gurjunene (**6**)					
Viridiflorol (**26**)					
Aromadendrene (**11**)	*Muscodor yucatanensis*		Muscodor		[39]
Spathulenol (**35**)	*Monascus purpureus*		Monascus		[40]

^a^ The components were mainly investigated via gas chromatography-mass spectrometry (GC/MS) in the literature.

**Table 2:** Aromadendrene-type STSs.

Name	ID	Products	Species	Ref.
MpMTPSL4	KU664191	*α*-Gurjunene (**17**)	*Marchantia polymorpha*	[60]
FhTPS8	Unigene_80141	*α*-Gurjunene (**17**)	*Freesia x hybrida*	[61]
PpSTP06	-	*α*-Gurjunene (**17**)	*Postpartum*	[62]
GbTPS1	GB_D01G0996	*α*-Gurjunene (**17**)	*Gossypium barbadense*	[63]
Agr2	A0A5Q0QNJ2	Viridiflorene (**18**)	*Agrocybe aegerita*	[38]
SLT18	BAP82213.1	Viridiflorene (**18**)	*Streptomyces lactacystinaeus*	[64]
SiTPS	JGI Protein Id: 77541	Viridiflorene (**18**); Viridiflorol (**26**)	*Serendipita indica*	[65]
Sav_76	BA000030.4	Avermitilol; Viridiflorol (**26**)	*Streptomyces avermitilis*	[66]
Agr5	A0A5Q0QSI8.1	Viridiflorol (**26**)	*Agrocybe aegerita*	[38]
CryA	A0A7W7FVV8	*ent*-Viridiflorol (**27**)	*Crossiella cryophila*	[67]
MqTPS1	-	Viridiflorol (**26**)	*Melaleuca quinquenervia*	[68]
MpTPS4	MH790402.1	Viridiflorol (**26**)	*Mentha piperita*	[10,69]
